# Prognostic Value of S100 Family mRNA Expression in Hepatocellular Carcinoma

**DOI:** 10.5152/tjg.2024.22658

**Published:** 2024-04-01

**Authors:** Renrui Wan, Zhenhua Tan, Hai Qian, Peng Li, Jian Zhang, Xiaofeng Zhu, Ping Xie, Lingyan Ren

**Affiliations:** 1 Department of Hepatobiliary Surgery, Huzhou Central Hospital, Zhejiang University Huzhou Hospital, Affiliated Central Hospital of Huzhou Teachers College, Huzhou, Zhejiang, China; 2 Department of Operating Room, Huzhou Central Hospital, Affiliated Central Hospital Huzhou Teachers College, Huzhou, Zhejiang, China; 3 Department of Nephrology, the First Affiliated Hospital of Huzhou Teachers College, the First People’s Hospital of Huzhou, Huzhou, Zhejiang, China

**Keywords:** S100, Hepatocellular Carcinoma, mRNA, Prognosis

## Abstract

**Background/Aims:**

The S100 family contains more than 20 Ca^2+^-binding proteins that participate in numerous cellular biological processes. However, the prognostic value of individual S100s in hepatocellular carcinoma (HCC) remains unclear. Therefore, we comprehensively assessed the prognostic value of S100s in HCC.

**Materials and Methods:**

The mRNA level of S100s in distinct types of cancer was analyzed through Oncomine. The clinical prognostic significance of each S100 was evaluated using Kaplan–Meier plotter and OncoLnc. The expression and mutation of S100s were determined through cBioPortal. Gene Ontology and Kyoto Encyclopedia of Genes and Genomes analyses were used to predict the functions and pathways of S100s.

**Results:**

The analyses revealed that, relative to normal tissues, liver cancer tissues showed aberrant mRNA expression of most S100s. In the survival analysis with Kaplan–Meier plotter, elevated expression levels of S100PBP, S100A2, S100A7, S100A10, and S100A13 were related to shorter overall survival (OS), whereas increased S100A5 expression was associated with longer OS. Moreover, results obtained using OncoLnc showed that increased expression levels of S100P, S100PBP, S100A13, S100A11, S100A10, and S100A2 were related to shorter OS. Thus, S100PBP, S100A13, S100A10, and S100A2 exhibited the same prognostic trend in the 2 databases. However, all S100 member gene mutational changes had no considerable prognostic value in OS and disease-free survival of HCC patients.

**Conclusion:**

Although the findings need to be further confirmed by experiments, they provide new evidence for the prognostic significance of the S100s in HCC.

**Main Points**

- We examined the expression profile of S100s in hepatocellular carcinoma (HCC) patients and determined the prognostic value of 21 members of S100 family for HCC through the Oncomine database, OncoLnc, Kaplan–Meier plotter, cBioPortal, Gene Ontology, and Kyoto Encyclopedia of Genes and Genomes.
- Compared with normal tissues, 15 members of the S100 family showed remarkable differences in their expression pattern in the HCC tissues, and 8 members were remarkably associated with HCC prognosis.
- Evaluation of the correlation between S100s and HCC with various clinical characteristics showed that distinct S100s may engage with diverse signaling pathways, assuming distinct roles in the pathophysiology of HCC.

## Introduction

According to the latest research, liver cancer ranks sixth in global cancer incidence and fourth in terms of leading causes of death. In 2020, liver cancer accounted for a total of 905 677 new cases and 830 180 deaths globally, with over half occurring in China.^1^ Hepatocellular carcinoma (HCC) is the predominant form of liver cancer, constituting 70%-80% of all diagnosed cases. It is well known that liver cancer has a poor prognosis, which is associated with numerous factors; moreover, in the case of HCC in particular, one of these factors is lack of effective indicators for prognostic monitoring of the cancer. Therefore, identifying effective prognostic biomarkers for HCC diagnosis and monitoring, as well as investigating cancer based on the identified biomarkers, will help improve HCC prognosis. 

The Ca^2+^-binding protein S100 was first reported in 1965, followed by the discovery of >20 members of the protein family since then.^2^ Some of the S100s have been extensively shown to play a pivotal role in the onset and progression of malignant tumors, and certain S100 family members have also recently been reported to be related to multiple tumors, such as ovarian and breast cancer.^3,4^ Therefore, the level of the S100s might predict the HCC grade and stage, although this possibility has not been reported to date. Thus, we used various databases to verify the relevance between S100s and HCC prognosis and thereby provide a reference for further studies on HCC.

## Materials and methods

### Oncomine Analysis

The Oncomine database (http://www.oncomine.org)^5^ was employed to analyze the mRNA expression of S100s in distinct cancers. To determine the difference, the S100 family mRNA expression in clinical tumor specimens was compared to the levels in corresponding normal tissues. The Student’s *t*-test was employed to calculate *P*-values. The subsequent criteria were established for each gene: *P* < .01, fold-change >2, and gene rank within the top 10%. 

### Kaplan–Meier Plotter Analysis

The researchers harnessed the Kaplan–Meier plotter database (www.kmplot.com) for an in-depth analysis of the association between S100s and the prognosis of HCC. This comprehensive database aggregates data from both Gene Expression Omnibus and The Cancer Genome Atlas (TCGA), encompassing gene expression profiles and prognostic insights across a diverse spectrum of tumors,^6^ Using this database, we assessed the prognostic significance of the S100 family in HCC. The researchers divide the samples into high- and low-expression groups by median expression to calculate the overall survival (OS) together with the hazard ratio (HR), 95% confidence intervals (95% CI), and log-rank *P* value; *P* < .05 was considered statistically significant.

### OncoLnc Database Analysis

To analyze the prognostic significance of the S100s in HCC, we employed OncoLnc (http://www.oncolnc.org/), ^7^ an online database that can correlate the TCGA survival data with the gene levels of various cancers. This database contained gene expression data (derived from liver tumor tissue) and survival information for 360 patients with clinically diagnosed HCC. The patient samples were again separated into low- and high-expression groups using the median level of S100s to calculate the OS together with the log-rank *P*-value. The following threshold was set for each gene: *P* = .05.

### cBioPortal and The Cancer Genome Atlas Analysis

We selected the HCC (TCGA Provisional, now known as TCGA, Firehose Legacy) data set containing 442 cases of pathology reports and gene expression information (derived from liver tumor tissue), and used cBioPortal (http://www.cbioportal.org),^8,9^ to analyze the relevance between S100s and HCC. We utilized the prognostic values from cBioPortal to determine both overall survival (OS) and disease-free survival (DFS). The significance threshold was set at *P* < .05.

### Gene Ontology and Kyoto Encyclopedia of Genes and Genomes *Analysis*

Utilizing comparative data sourced from TCGA (https://portal.gdc.cancer.gov/) encompassing HCC and normal liver tissues (there were 50 cases of normal tissue and 374 cases of HCC), we conducted a search for differentially expressed S100 genes. Among these selected S100 genes, we employed the cor.test function in R language (version 4.8.2) to identify the top 50 most pertinent genes. Subsequently, we performed Kyoto Encyclopedia of Genes and Genomes (KEGG) and Gene Ontology (GO) enrichment analyses using the clusterProfiler R package (version 4.8.2), aiming to discern notably enriched functions and pathways. *P* < .05 was chosen as the cutoff criteria.^10^

## Results

### The mRNA Level of S100s in Different Cancers

**Figure 1.**
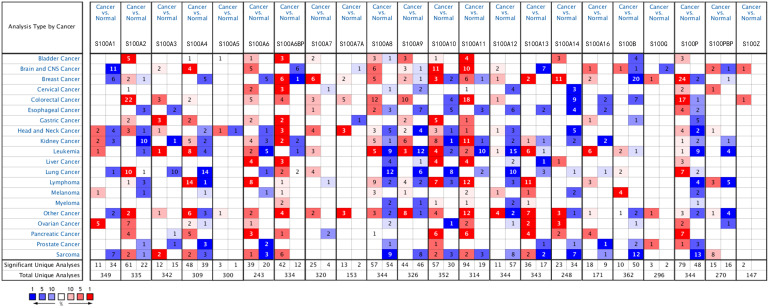
The transcription levels of S100 members in different types of cancer. This graphic was generated from Oncomine, indicating the numbers of datasets with statistically significant (*P* < .01) mRNA overexpression (red) or underexpression (blue) of S100 members (different types of cancer vs. corresponding normal tissue). The number in the colored cell represents the number of analyses meeting thresholds. Cell color is determined by gene rank. The more intense red (overexpression) or blue (underexpression) indicates a more highly significant overexpressed or underexpressed gene.

We first used Oncomine to assess variations in the mRNA expression profile of S100s across multiple cancers between tumor specimens and normal tissues. The database contained 349, 335, 342, 309, 300, 243, 334, 153, 320, 344, 326, 352, 314, 344, 343, 248, 171, 362, 296, 270, 344, and 147 analyses for S100Z, S100A1, S100A2, S100B, S100A3, S100A4, S100A5, S100A6BP, S100A6, S100A7, S100A7A, S100A8, S100A9, S100A10, S100A11, S100A12, S100A13, S100A14, S100A16, S100PBP, S100P, and S100G, respectively (Figure 1). While the mRNAs encoding S100P, S100A6, S100A6BP, S100A10, and S100A11 were overexpressed in HCC patients, the mRNA levels of S100A8, S100A9, and S100A12 were found to be downregulated.

Next, oncomine was employed to analyze the differential expression of S100s between normal tissues and HCC (Table 1). In the Roessler Liver 2 dataset,^11^ we found an increased mRNA expression of S100P, S100A6BP, and S100A10 in HCC, and in the Roessler Liver dataset,^11^ we detected elevated mRNA expression of S100P, S100A6, S100A6BP, S100A10, and S100A11 in HCC tissues relative to normal tissues. In the Chen et al^12^ dataset, S100P and S100A8 mRNA levels were higher and lower, respectively, in HCC than in normal tissues. In the Mas et al^13^ dataset, the mRNA expression of S100A6, S100A10, and S100A11 was upregulated, but that of S100A8 and S100A12 was downregulated in HCC. In the Wurmbach et al^14^ dataset, S100A6BP mRNA level was upregulated, whereas the mRNA expression of S100A8, S100A9, and S100A12 was downregulated. Other S100 members showed no significant differences between tumor and normal tissues.

**Figure 2.**
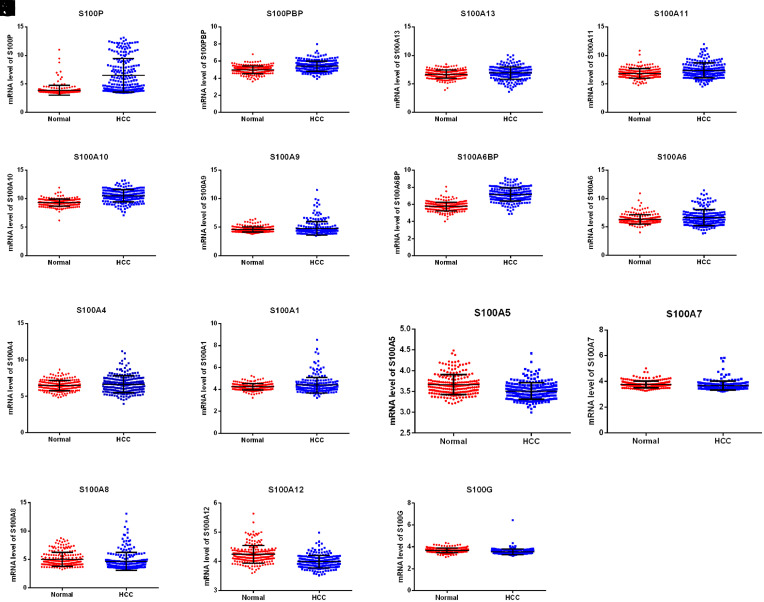
The differential expression of S100 family members in normal tissues and hepatocellular carcinoma tissues. *P* < .05 (Oncomine database).

Lastly, Oncomine was employed to examine the genetic activity of S100 family members that showed differential expression between normal and HCC tissues in the Roessler Liver 2 dataset, which included the largest number of samples. In HCC tissues, the mRNA levels of S100A1, S100A4, S100A6, S100A6BP, S100A9, S100A10, S100A11, S100A13, S100P, and S100PBP were upregulated, while the mRNA levels of S100G, S100A5, S100A7, S100A8 and S100A12 were downregulated (Figure 2). In this dataset, other S100 family members showed no remarkable expression differences between the normal and tumor tissues.

### Prognostic Value of S100 in Hepatocellular Carcinoma Patients

**Figure 3.**
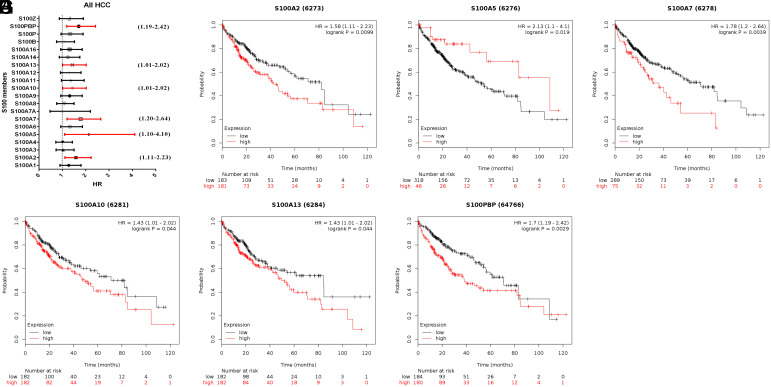
The prognostic value of mRNA level of S100 members in HCC patients (OS in Kaplan–Meier plotter). (A) Prognostic HRs of individual S100 members in all HCC. (B-G) Survival curves of S100A2 (RNA-Seq ID: 6273), S100A5 (RNA-Seq ID: 6276), S100A7 (RNA-Seq ID: 6278), S100A10 (RNA-Seq ID: 6281), S100A13 (RNA-Seq ID: 6284), S100PBP (RNA-Seq ID: 64766). *P* < .05. HCC, hepatocellular carcinoma; HR, hazard ratio; OS, overall survival.

**Figure 4.**
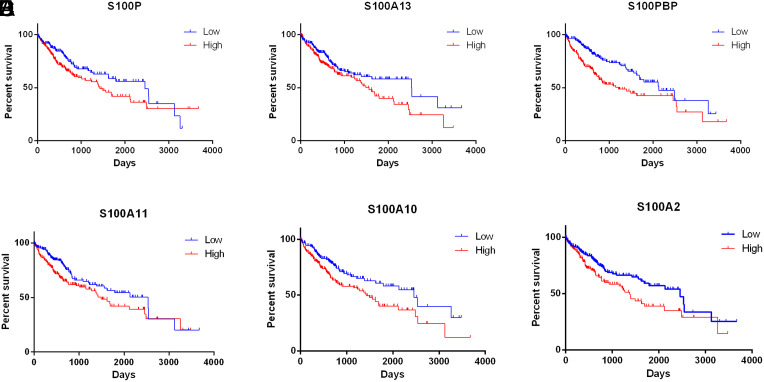
The prognostic value of mRNA level of S100 members in hepatocellular carcinoma patients (OS in OncoLnc. (A-F) Survival curves of S100P, S100A13, S100PBP, S100A11, S100A10 and S100A2. *P* < .05. OS, overall survival.

We employed Kaplan–Meier plotter and OncoLnc to investigate whether the prognosis of HCC patients is related to the S100 family. Specifically, we calculated the OS associated with each gene individually. In the Kaplan–Meier plotter analysis, increased S100PBP, S100A2, S100A7, S100A10, and S100A13 expression levels were related to shorter OS (Figure 3B and 3D-G), whereas elevated S100A5 expression was associated with longer OS (Figure 3C). In the OncoLnc analysis, elevated expression of S100P, S100PBP, S100A13, S100A11, S100A10, and S100A2 was related to shorter OS (Figure 4A-F).

### Prognostic Value of S100s in Varying Clinical States of Hepatocellular Carcinoma Patients

**Figure 5.**
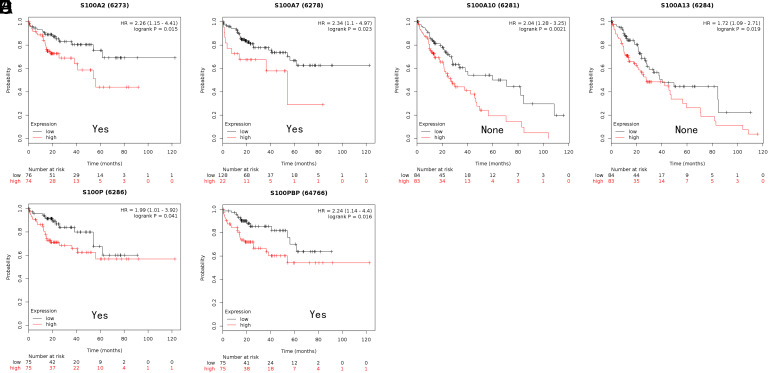
Correlation of S100 members with differential hepatitis virus status of hepatocellular carcinoma patients.

**Figure 6.**
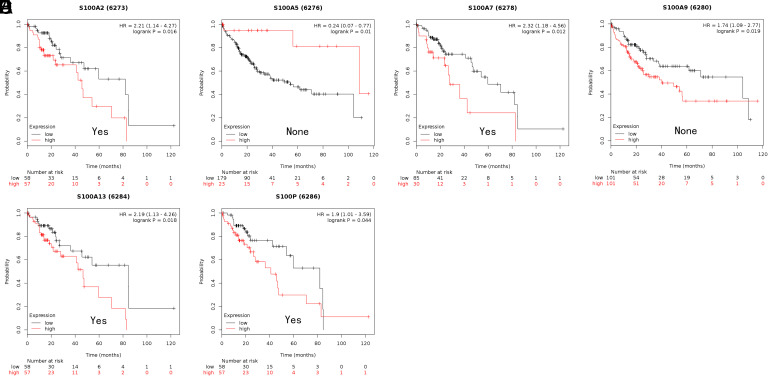
Correlation of S100 members with differential alcohol consumption status of hepatocellular carcinoma patients.

**Figure 7.**
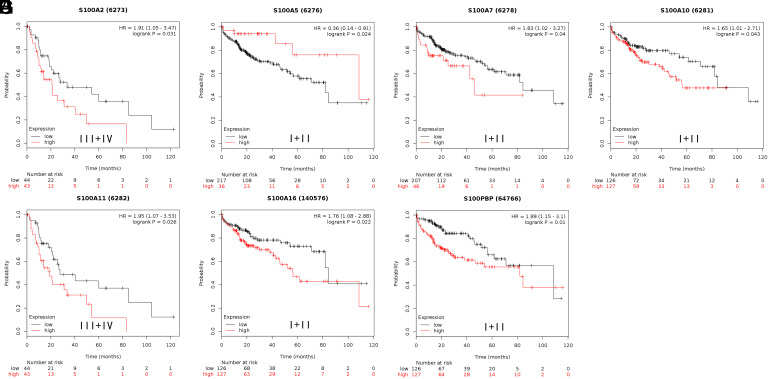
Correlation of S100 members with differential clinical stage status of hepatocellular carcinoma patients.

**Figure 8.**
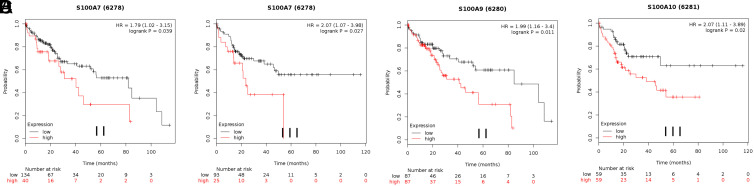
Correlation of S100 members with different pathological grade status of hepatocellular carcinoma patients.

**Figure 9.**
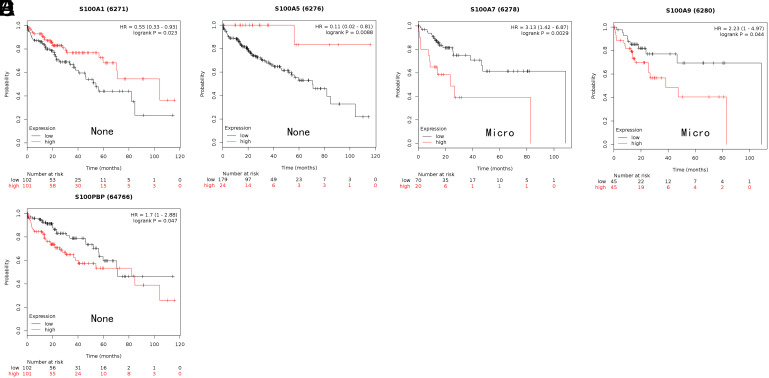
Correlation of S100 members with different vascular invasion status of hepatocellular carcinoma patients.

We next ascertained the prognostic significance of S100s in HCC patients with respect to clinical status; specifically, our analysis included hepatitis virus infection, history of alcohol consumption (alcohol abuse), HCC clinical stage, pathological grade of cancer, and vascular invasion (Figures 5–9). 

First, in HCC patients with hepatitis virus infection, elevated mRNA levels of S100A2, S100A7, S100P, and S100PBP correlated with reduced OS, whereas in HCC patients without hepatitis virus infection, elevated mRNA levels of S100A10 and S100A13 were relevant to shorter OS (Figure 5). The expression levels of the remaining S100s did not differ remarkably. The detailed prognostic results are presented in Table 2.

Second, in HCC patients with a history of alcohol abuse, elevated expressions of S100A2, S100A7, S100A13, and S100P were related to shorter OS, but in HCC patients without a history of alcohol abuse, elevated expressions of S100A9 and S100A5 were associated with shorter OS and longer OS, respectively (Figure 6). Other S100 family members did not show any considerable difference in the expression profile. Table 3 lists all calculated prognostic values.

Third, in patients with clinical stage III+IV HCC, elevated expressions of S100A2 and S100A11 correlated with shorter OS. Shorter OS was also observed in patients with clinical stage I+II HCC in whom the expressions of S100A7, S100A10, S100A16, and S100PBP were upregulated. Longer OS was observed in patients with clinical stage I+II HCC in whom S100A5 expression was upregulated (Figure 7). Other members of the S100 family showed no considerable differences in expression. The detailed prognostic results are presented in Table 4.

Fourth, when the HCC cases were categorized according to cancer pathological grade (Figure 8), high mRNA expression of S100A7 in pathological grades II and III correlated with shorter OS, as did high expression of S100A9 in pathological grade II and of S100A10 in pathological grade III. Other S100 family members exhibited no considerable differences in expression. Table 5 contains the complete prognostic results.

Fifth, in HCC patients with microvascular invasion, elevated expression of S100A7 and S100A9 was associated with shorter OS, whereas in HCC patients with no vascular invasion, high expression of S100A1 and S100A5 correlated with longer OS but elevated expression of S100PBP was linked to shorter OS (Figure 9). Other members of the S100 family showed no considerable differences in expression. Table 6 shows the results in detail.

### Alteration of S100 Family Genes Does Not Affect Disease-Free Survival and Overall Survival in Hepatocellular Carcinoma Patients

**Figure 10.**
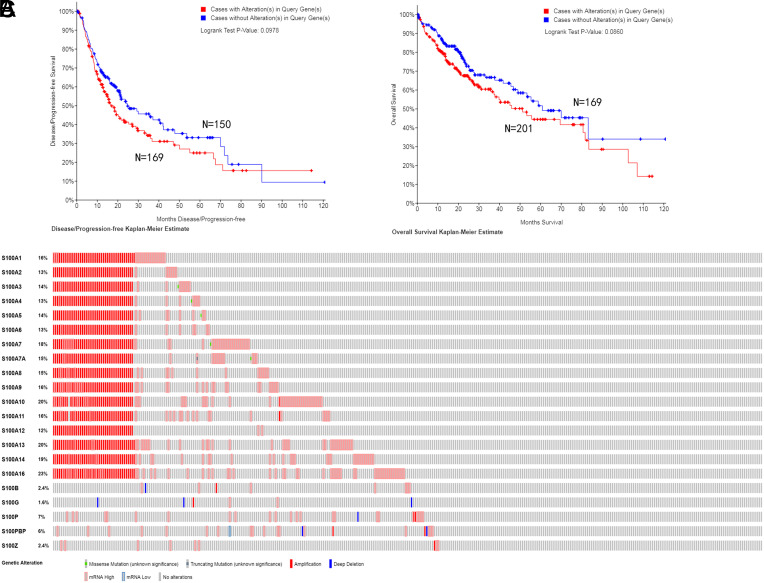
S100 family genes’ expression and mutation analysis in HCC (cBioPortal). (A) Kaplan–Meier plots comparing disease-free survival in cases with/without S100 alterations. (B) Kaplan–Meier plots comparing overall survival in cases with/without S100 alterations. (C) Oncoprint in cBioPortal represented the proportion and distribution of samples with alterations in S100 factors. The figure was cropped on the right to exclude samples without alterations. HCC, hepatocellular carcinoma.

S100 family genes were altered in 201/360 samples of patients with HCC (55.8%). All S100 family genes exhibited amplification changes (Figure 10C). The log-rank test and Kaplan–Meier survival analysis results showed that the alterations of S100 family genes did not differ considerably with respect to DFS and OS in HCC patients carrying or not carrying S100 family gene alterations (Figure 10A). 

### The Functions and Pathways Prediction of Abnormal Expression of S100s and the Related Genes in Hepatocellular Carcinoma Patients

**Figure 11.**
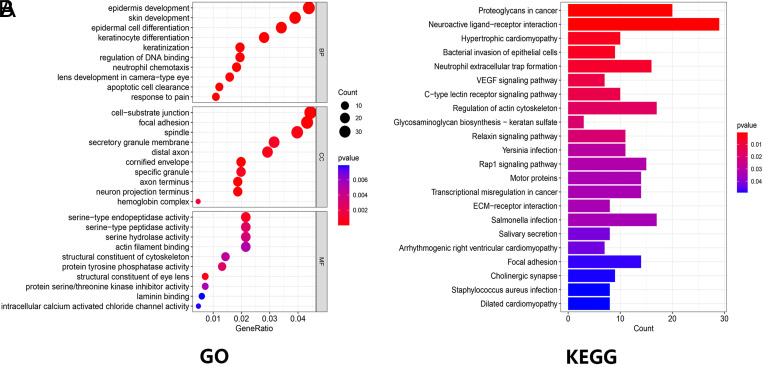
The function and pathway enrichment analysis of S100s and genes remarkably associated with S100s alterations by GO and KEGG. (A) GO functional enrichment analysis predicted 3 main functions, including biological process, cellular components, and molecular functions. (B) KEGG pathway enrichment analysis. GO, Gene Ontology; KEGG, Kyoto Encyclopedia of Genes and Genomes.

Through GO analysis, as depicted in Figure 11A, we observed significant regulation in various biological processes, namely epidermis development, epidermal cell differentiation, skin development, and keratinocyte differentiation. Additionally, alterations in S100s demonstrated noteworthy influence on cellular components, encompassing cell-substrate junctions, focal adhesions, and spindles. Furthermore, molecular functions such as serine-type endopeptidase activity, serine-type peptidase activity, and serine hydrolase activity exhibited marked regulation by the S100s alteration in HCC. Further employing KEGG analysis, we identified that the shifts in S100s expression were intricately linked to several pathways, including proteoglycans in cancer, neuroactive ligand-receptor interaction, and neutrophil extracellular trap formation (Figure 11B).

## Discussion

Numerous previous studies on the molecular mechanisms underlying the etiology and prognosis of HCC have been widely questioned due to the insufficient sample size. Many studies have reported that the S100 family is involved in the complex biological processes of diverse diseases, such as the occurrence and development of tumor cells, drug resistance, and heart failure.^15^ However, there are still many unknowns about the specific molecular mechanism of the S100 protein involved in the pathophysiology of HCC. Our endeavor focused on delving into the expression, mutations, and correlation between S100s and HCC prognosis using extensive data sets from various databases. Consequently, this study holds significant reference value for advancing research on the molecular mechanisms underlying HCC.

In the present study, we investigated the changes in the expression level of S100s and their relationship with the prognosis of HCC. We found that the expression levels of S100PBP, S100A2, S100A7, S100A10, S100A13, S100A5, S100P, and S100A11 were considerably correlated with prognosis, although only S100A5 expression was remarkably relevant to superior prognosis. By comparing the results obtained using distinct databases, we determined that S100PBP, S100A13, S100A10, and S100A2 presented the same prognostic trend in the Kaplan–Meier plotter and OncoLnc analyses. These findings suggest that S100PBP, S100A13, S100A10, and S100A2 hold the potential to emerge as biomarkers for the prognosis or treatment of HCC.

Based on the analysis conducted through the cBioPortal online tool, S100s displayed an overall mutation rate of 55.8% in HCC. However, the prognostic analysis did not conclusively establish a clear association between S100s and HCC prognosis. This finding diverges from the research outcomes of Zheng et al.^16^ One possible explanation could be attributed to the fact that S100s encompasses more than 20 subgenes, and each gene’s influence on HCC may potentially be interdependent or restrictive. As such, its specific mechanism warrants further investigation. Fortunately, the results of the GO and KEGG enrichment analyses offer valuable insights for further exploration into the molecular mechanisms.

S100A2 has been widely demonstrated to play a part in promoting tumor progression, such as in pancreatic adenocarcinoma^17^ and esophageal carcinoma.^18^ Nevertheless, research has revealed that S100A2 exhibits anti-cancer properties in gastric cancer.^19^ The precise reasons behind the divergent effects of the same gene in distinct tumor types remain elusive, underscoring the intricate nature of tumor molecular regulatory networks. Consequently, the molecular mechanisms underlying this phenomenon warrant further in-depth investigation.

Yan et al^20^ discovered that elevated S100A2 expression was linked to adverse prognosis and clinicopathological features in HCC. Moreover, inhibition of S100A2 gene expression can delay the proliferation and metastasis of HepG2 cell line. Our research results are consistent with those of Yan et al.^20^ The prognostic analysis conducted through Kaplan–Meier plotter and OncoLnc revealed that the elevated expression of S100A2 mRNA correlated with a shorter OS. However, our research revealed that the mRNA expression of S100A2 in HCC was within normal levels. One plausible explanation is that the tumor microenvironment could elevate the protein expression of certain genes, consequently influencing the prognosis. This hypothesis necessitates further investigation for validation.

Study found a significant role for S100A10 in diversified malignant tumors. Li et al^21^ elaborated that S100A10 is markedly upregulated in gastric cancer and activates the mTOR pathway by interacting with annexin A2 to accelerate tumor glycolysis, thereby promoting malignant cell proliferation while suppressing apoptosis. Yanagi et al^22^ found that S100A10 acts as a promoter of breast cancer stem cells, thereby increasing the invasion ability of breast cancer. Zhou et al^23^ found that upregulation of S100A10 can increase the proliferation of Hep3B and Huh-7 HCC cell lines, while the cell viability of S100A10 knockout cell lines was significantly reduced, and animal studies have shown similar results. Wang et al^24^ found that S100A10 can act on signaling such as AKT and ERK through extracellular vesicle secretion, thereby accelerating the proliferation and metastasis of HCC. Our research found that S100A10 was elevated expressed in HCC and predicted poor prognosis. The results are consistent with the above research reports, and they can support each other.

S100A13, a relatively newly identified S100 family member, is characterized by the strong specificity of its expression in multiple tumors. Miao et al^25^ reported that elevated S100A13 level is linked to tumor angiogenesis in patients in the early-stage of non-small cell lung cancer, leading to poor prognosis. Moreover, ectopic expression of S100A13 was previously shown to increase tumor growth in thyroid cancer through HMGA1.^26^ Our study demonstrated upregulated S100A13 in HCC, and high expression of S100A13 predicts shorter OS. This aligns with Peng et al’s^27^ research findings.

Studies have found that S100PBP is a binding protein of S100P and has no homology with existing S100s proteins. Lines et al^28^ reported that in pancreatic ductal adenocarcinoma and metastatic lesions, S100PBP expression is notably diminished and that the adhesion of tumor cells is markedly reduced after S100PBP upregulation and increased after S100PBP silencing. Lu et al^29^ found that S100PBP may inhibit pancreatic cancer cell adhesion through the LMNB1 and PRKRA gene pathways. However, studies on the relationship between S100PBP and HCC are rare. We found that S100PBP is upregulated in HCC and is linked to a less favorable prognosis. This study serves as a valuable complement to understanding the association between S100PBP and HCC prognosis.

Based on existing studies, it is found that the abnormal expression of S100P is associated with a variety of tumors, and increased S100P expression is related to poor prognosis.^30,31^ Cong et al^32^ found that in breast cancer, S100P promotes tumor progression but enhances chemosensitivity. Qi et al^33^ noted that heightened S100P expression in HCC strongly correlates with portal vein tumor thrombus and microvascular invasion, highlighting its potential as a therapeutic target for HCC metastasis. We found that in patients with HCC, S100P expression was increased, and the results of prognostic analysis performed using OncoLnc showed that high S100P expression was related to shorter OS, although similar findings were not obtained using Kaplan–Meier plotter.

Aberrant expression of S100A11 was found to be correlated with diverse tumors. Meng et al^34^ reported that S100A11 can enhance the proliferation and invasion of cervical cancer cells. Tu et al^35^ found that S100A11 acts as an oncogene in glioblastoma through the S100A11/ANXA2/NF-κB positive feedback loop. Sobolewski et al^36^ reported that S100A11/ANXA2 belongs to a tumor oncogene/tumor suppressor gene network, which can be deregulated through steatosis at an early stage, thereby participating in the development of inflammation and HCC. We showed that S100A11 expression was markedly elevated in patients with HCC compared to healthy controls. Prognostic analysis performed using OncoLnc revealed that high S100A11 expression was related to shorter OS, although similar findings were not obtained using Kaplan–Meier plotter.

Our study had a few limitations. First, our study only illustrates the difference between patients with HCC and healthy controls with respect to the S100 family; the molecular mechanisms underlying the relationship between the S100 family and HCC were not investigated, and thus additional studies are required to determine the mechanisms involved. Second, because of the limitations of the data, we could not perform multivariate analysis by using logistic or COX regression. Therefore, the patient survival associated with S100 family members according to their specific expression levels might be influenced by multiple factors, including classification and staging. However, this effect was not reflected in our analysis, and thus additional comprehensive research must be conducted to address this matter.

We analyzed the expression profile of S100s in HCC patients and evaluated the prognostic significance of 21 members of S100s through the Oncomine database, OncoLnc, Kaplan–Meier plotter, cBioPortal, GO, and KEGG. Compared with normal tissues, 15 members of the S100s showed remarkable differences in their expression pattern in the HCC tissues, and among them, 8 were remarkably correlated with the prognosis of HCC. Further evaluation of the correlation between S100s and HCC with multiple clinical statuses showed that some S100 family members may interact with a variety of different signaling pathways and significantly contribute to the pathophysiology of HCC.

The study’s findings establish a foundational basis for further investigation of the mechanistic link between S100 family members and the development of HCC, along with the regulation of distinct signaling pathways in HCC. Our study also presents a potential novel prognostic biomarker for liver cancer and could promote the development of targeted therapies for liver cancer in the future.

## Data Availability

The data used and/or analyzed during the current study were from online public databases and are available from the corresponding author upon reasonable request.

## Figures and Tables

**Table 1.** Significant Changes of S100 Members’ Expression in Transcription Level Between Hepatocellular Carcinoma and Normal Liver Tissues (Oncomine Database)

Gene	Dataset	Normal (Cases)	Tumor (Cases)	Fold Change	*t*-Test	*P*
S100P	Chen	Liver (74)	HCC (101)	3.440	6.353	1.26 × 10^−09^
	Roessler Liver	Liver (21)	HCC (22)	3.614	2.600	.007
	Roessler Liver 2	Liver (220)	HCC (225)	6.199	12.941	2.69 × 10^−30^
S100A6	Mas	Liver (19)	HCC (38)	3.111	9.680	8.84 × 10^−14^
	Roessler Liver	Liver (21)	HCC (22)	2.823	3.502	9.16 × 10^−04^
S100A6BP	Roessler Liver 2	Liver (220)	HCC (225)	2.663	22.142	1.27 × 10^−71^
	Roessler Liver	Liver (21)	HCC (22)	2.125	7.595	2.67 × 10^−09^
	Wurmbach	Liver (10)	HCC (35)	2.247	6.216	7.17 × 10^−07^
S100A8	Chen	Liver (76)	HCC (104)	−2.672	−7.602	8.22 × 10^−13^
	Mas	Liver (19)	HCC (38)	−3.959	−6.089	9.31 × 10^−08^
	Wurmbach	Liver (10)	HCC (35)	−14.197	−5.235	.0000338
S100A9	Wurmbach	Liver (10)	HCC (35)	−4.426	−2.839	.007
S100A10	Mas	Liver (19)	HCC (38)	2.201	9.544	3.38 × 10^−12^
	Roessler Liver 2	Liver (220)	HCC (225)	2.341	14.830	1.60 × 10^−39^
	Roessler Liver	Liver (21)	HCC (22)	2.209	4.079	1.27 × 10^−04^
S100A11	Mas	Liver (19)	HCC (38)	2.878	9.396	9.50 × 10^−13^
	Roessler Liver	Liver (21)	HCC (22)	2.067	3.58	6.01 × 10^−04^
S100A12	Mas	Liver (19)	HCC (38)	−2.347	−4.82	.0000616
	Wurmbach	Liver (10)	HCC (35)	−6.383	−3.141	.006

HCC, hepatocellular carcinoma.

**Table 2.** Prognostic Value of S100 Family Members in Hepatocellular Carcinoma Patients With and Without Hepatitis Virus Infection

S100 Family	RNA-Seq ID	Number of Patients (n)		Hepatitis virus	HR	95% CI	*P*
		Low Expression	High Expression				
S100A1	6271	75	75	Yes	0.87	0.45-1.66	.67
		84	83	None	0.81	0.51-1.27	.36
S100A2	6273	76	74	Yes	2.26	1.15-4.41	.015
		86	81	None	0.94	0.6-1.47	.78
S100A3	6274	75	75	Yes	1.53	0.79-2.95	.21
		89	78	None	0.63	0.4-1.01	.051
S100A4	6275	75	75	Yes	0.79	0.41-1.52	.48
		84	83	None	0.86	0.55-1.34	.5
S100A5	6276	132	18	Yes	0.19	0.03-1.36	.063
		140	27	None	0.5	0.24-1.01	.047
S100A6	6277	75	75	Yes	1.43	0.74-2.75	.28
		84	83	None	1.04	0.67-1.63	.85
S100A7	6278	128	22	Yes	2.34	1.1-4.97	.023
		125	42	None	1.62	0.96-2.75	.068
S100A7A	338824	147	3	Yes	3.05	0.73-12.73	.11
		158	9	None	0.7	0.22-2.24	.55
S100A8	6279	75	75	Yes	1.03	0.54-1.97	.93
		84	83	None	0.73	0.46-1.15	.18
S100A9	6280	75	75	Yes	1.77	0.92-3.43	.085
		84	83	None	1.1	0.7-1.72	.68
S100A10	6281	75	75	Yes	1.32	0.69-2.52	.41
		84	83	None	2.04	1.28-3.25	.0021
S100A11	6282	75	75	Yes	1.4	0.73-2.68	.31
		84	83	None	1.16	0.74-1.82	.53
S100A12	6283	82	68	Yes	0.91	0.47-1.75	.78
		84	83	None	0.66	0.42-1.05	.075
S100A13	6284	75	75	Yes	1.23	0.64-2.35	.53
		84	83	None	1.72	1.09-2.71	.019
S100A14	57402	75	75	Yes	0.84	0.44-1.6	.59
		84	83	None	1.21	0.77-1.89	.4
S100A16	140576	75	75	Yes	1.41	0.74-2.71	.29
		84	83	None	1.47	0.94-2.31	.092
S100B	6285	76	74	Yes	1.35	0.7-2.59	.36
		86	81	None	0.92	0.58-1.44	.7
S100G	795	—	—	—	—	—	—
		—	—	—	—	—	—
S100P	6286	75	75	Yes	1.99	1.01-3.92	.041
		85	82	None	1.36	0.87-2.13	.18
S100PBP	64766	75	75	Yes	2.24	1.14-4.4	.016
		84	83	None	1.29	1.82-2.02	.28
S100Z	170591	79	71	Yes	0.64	0.33-1.25	.19
		110	57	None	0.61	0.36-1.02	.057

*P* < .05 was considered to be statistically significant.

HR, hazard ratio.

**Table 3.** Prognostic Value of S100 Family Members in Hepatocellular Carcinoma Patients With and Without History of Alcohol Abuse

S100 Family	RNA-Seq ID	Number of Patients (n)		Alcohol Consumption	HR	95% CI	*P*
		Low Expression	High Expression				
S100A1	6271	59	56	Yes	1.28	0.67-2.45	.44
		101	101	None	0.72	0.45-1.15	.17
S100A2	6273	58	57	Yes	2.21	1.14-4.27	.016
		103	99	None	1.44	0.91-2.28	.12
S100A3	6274	58	57	Yes	1.09	0.58-2.06	.79
		102	100	None	0.95	0.6-1.51	.84
S100A4	6275	58	57	Yes	1.04	0.55-1.97	.91
		102	100	None	0.71	0.45-1.13	.15
S100A5	6276	93	22	Yes	0.87	0.38-1.98	.75
		179	23	None	0.24	0.07-0.77	.01
S100A6	6277	58	57	Yes	1.82	0.95-3.91	.069
		101	101	None	1.13	0.72-1.79	.59
S100A7	6278	85	30	Yes	2.32	1.18-4.56	.012
		168	34	None	1.7	0.96-3.02	.065
S100A7A	338824	112	3	Yes	1.11	0.15-8.19	.92
		193	9	None	1.24	0.45-3.41	.68
S100A8	6279	59	56	Yes	0.82	0.44-1.55	.54
		102	100	None	1.08	0.68-1.7	.75
S100A9	6280	58	57	Yes	1.4	0.74-2.66	.3
		101	101	None	1.74	1.09-2.77	.019
S100A10	6281	58	57	Yes	1.36	0.72-2.57	.34
		101	101	None	1.35	0.85-2.14	.21
S100A11	6282	58	57	Yes	1.4	0.74-2.66	.3
		101	101	None	1.28	0.81-2.04	.29
S100A12	6283	70	45	Yes	0.55	0.27-1.1	.087
		111	91	None	0.9	0.57-1.43	.65
S100A13	6284	58	57	Yes	2.19	1.13-4.26	.018
		101	101	None	1.25	0.79-1.97	.34
S100A14	57402	58	57	Yes	1.73	0.9-3.31	.096
		101	101	None	1.03	0.65-1.62	.91
S100A16	140576	58	57	Yes	1.64	0.85-3.15	.13
		101	101	None	1.16	0.73-1.83	.53
S100B	6285	59	56	Yes	0.68	0.36-1.32	.25
		103	99	None	1.22	0.77-1.93	.4
S100G	795	—	—	—	—	—	
		—	—	—	—	—	
S100P	6286	58	57	Yes	1.9	1.01-3.59	.044
		101	101	None	1.3	0.82-2.06	.26
S100PBP	64766	59	56	Yes	1.72	0.89-3.31	.1
		102	100	None	1.54	0.97-2.46	.065
S100Z	170591	60	55	Yes	0.62	0.33-1.09	.15
		146	56	None	0.7	0.4-1.21	.2

*P* < .05 was considered to be statistically significant.

HR, hazard ratio.

**Table 4.** Prognostic Value of S100 Family in Patients with Hepatocellular Carcinoma of Distinct Clinical Stages

S100 Family	RNA-Seq ID	Number of Patients (n)		Stage	HR	95% CI	*P*
		Low Expression	High Expression				
S100A1	6271	126	127	I + II	0.67	0.41-1.08	.099
		45	42	III + IV	1.08	0.61-1.9	.80
S100A2	6273	127	126	I + II	1.49	0.92-2.41	.1
		44	43	III + IV	1.91	1.05-3.47	.031
S100A3	6274	129	124	I + II	1.15	0.71-1.87	.56
		48	39	III + IV	1.06	0.59-1.9	.84
S100A4	6275	126	127	I + II	1.04	0.64-1.67	.89
		44	43	III + IV	1.22	0.69-2.16	.49
S100A5	6276	217	36	I + II	0.36	0.14-0.91	.024
		79	8	III + IV	1.51	0.59-3.87	.38
S100A6	6277	126	127	I + II	1.39	0.86-2.26	.17
		44	43	III + IV	1.42	0.79-2.56	.24
S100A7	6278	207	46	I + II	1.83	1.02-3.27	.04
		63	24	III + IV	1.36	0.74-2.5	.32
S100A7A	338824	241	12	I + II	0.93	0.29-2.96	.9
		82	5	III + IV	0.96	0.34-2.7	.94
S100A8	6279	126	127	I + II	0.78	0.48-1.27	.32
		44	43	III + IV	1.56	0.87-2.81	.13
S100A9	6280	126	127	I + II	1.54	0.95-2.51	.076
		44	43	III + IV	1.43	0.79-2.57	.23
S100A10	6281	126	127	I + II	1.65	1.01-2.71	.043
		44	43	III + IV	1.7	0.94-3.08	.077
S100A11	6282	126	127	I + II	1.36	0.84-2.2	.22
		44	43	III + IV	1.95	1.07-3.53	.026
S100A12	6283	139	114	I + II	0.62	0.38-1.02	.057
		45	42	III + IV	0.87	0.49-1.54	.62
S100A13	6284	126	127	I + II	1.43	0.88-2.31	.14
		44	43	III + IV	1.68	0.95-2.99	.073
S100A14	57402	126	127	I + II	1.04	0.65-1.68	.86
		44	43	III + IV	1.48	0.84-2.62	.18
S100A16	140576	126	127	I + II	1.76	1.08-2.88	.022
		44	43	III + IV	1.3	0.73-2.32	.37
S100B	6285	123	120	I + II	1.36	0.84-2.2	.21
		47	40	III + IV	0.9	0.5-1.64	.74
S100G	795	—	—	I + II	—	—	—
		—	—	III + IV	—	—	—
S100P	6286	126	127	I + II	1.28	0.79-2.07	.32
		44	43	III + IV	1.36	0.77-2.41	.28
S100PBP	64766	126	127	I + II	1.89	1.15-3.1	.01
		44	43	III + IV	1.12	0.63-1.97	.71
S100Z	170591	126	127	I + II	0.79	0.49-1.27	.33
		58	29	III + IV	0.78	0.41-1.49	.45

*P* < .05 was considered to be statistically significant.

HR, hazard ratio.

**Table 5.** Prognostic Value of S100 Family in Patients with Hepatocellular Carcinoma of Distinct Pathological Grades

S100 Family	RNA-Seq ID	Number of Patients (n)		Grade	HR	95% CI	*P*
		Low Expression	High Expression				
S100A1	6271	29	26	I	0.88	0.35-2.24	.79
		87	87	II	0.94	0.56-1.56	.8
		59	59	III	0.73	0.4-1.33	.29
		—	—	IV	—	—	—
S100A2	6273	28	27	I	1.49	0.59-3.79	.4
		90	86	II	1.33	0.79-2.21	.28
		66	52	III	1.73	0.95-3.15	.071
		—	—	IV	—	—	—
S100A3	6274	29	26	I	0.91	0.34-2.44	.85
		90	84	II	0.75	0.44-1.27	.28
		63	55	III	1.38	0.76-2.52	.29
		—	—	IV	—	—	—
S100A4	6275	28	27	I	1.06	0.42-2.67	.91
		87	87	II	1.21	0.73-2.02	.46
		59	59	III	0.82	0.45-1.49	.51
		—	—	IV	—	—	—
S100A5	6276	49	6	I	0.66	0.09-5.02	.68
		151	23	II	0.6	0.27-1.34	.21
		102	16	III	0.28	0.07-1.17	.063
		—	—	IV	—	—	—
S100A6	6277	28	27	I	1.14	0.45-2.92	.78
		87	87	II	1.34	0.8-2.23	.26
		59	59	III	1.16	0.64-2.1	.63
		—	—	IV	—	—	—
S100A7	6278	45	10	I	1.14	0.33-3.98	.84
		134	40	II	1.79	1.02-3.15	.039
		93	25	III	2.07	1.07-3.98	.027
		—	—	IV	—	—	—
S100A7A	338824	—	—	I	—	—	—
		166	8	II	1.09	0.39-3.04	.86
		111	7	III	1.04	0.32-3.37	.94
		—	—	IV	—	—	—
S100A8	6279	28	27	I	0.5	0.19-1.31	.15
		88	86	II	1.11	0.67-1.84	.69
		60	58	III	1.37	0.75-2.5	.3
		—	—	IV	—	—	—
S100A9	6280	28	27	I	0.4	0.15-1.07	.058
		87	87	II	1.99	1.16-3.4	.011
		59	59	III	1.38	0.75-2.52	.3
		—	—	IV	—	—	—
S100A10	6281	28	27	I	1.4	0.55-3.56	.48
		87	87	II	1.48	0.88-2.48	.14
		59	59	III	2.07	1.11-3.89	.02
		—	—	IV	—	—	—
S100A11	6282	28	27	I	0.79	0.3-2.07	.64
		87	87	II	1.51	0.9-2.52	.12
		59	59	III	1.58	0.86-2.92	.14
		—	—	IV	—	—	—
S100A12	6283	31	24	I	0.65	0.25-1.69	.38
		92	82	II	0.83	0.5-1.38	.48
		63	55	III	0.83	0.45-1.52	.54
		—	—	IV	—	—	—
S100A13	6284	28	27	I	0.72	0.28-1.83	.49
		87	87	II	1.42	0.85-2.38	.18
		59	59	III	1.31	0.72-2.39	.37
		—	—	IV	—	—	—
S100A14	57402	28	27	I	1.28	0.5-3.28	.6
		87	87	II	0.84	0.5-1.4	.5
		59	59	III	0.86	0.47-1.56	.61
		—	—	IV	—	—	—
S100A16	140576	28	27	I	1.02	0.4-2.58	.97
		87	87	II	1.18	0.71-1.97	.52
		59	59	III	1.26	0.69-2.3	.45
		—	—	IV	—	—	—
S100B	6285	29	26	I	0.9	0.35-2.29	.82
		96	78	II	0.98	0.58-1.66	.95
		63	55	III	1.13	0.62-2.06	.68
		—	—	IV	—	—	—
S100G	795	—	—	I	—	—	—
		—	—	II	—	—	—
		—	—	III	—	—	—
		—	—	IV	—	—	—
S100P	6286	28	27	I	0.8	0.31-2.04	.64
		87	87	II	1.11	0.67-1.85	.68
		59	59	III	1.61	0.87-2.95	.12
		—	—	IV	—	—	—
S100PBP	64766	28	27	I	2.35	0.89-6.25	.077
		88	86	II	1.64	0.97-2.75	.06
		59	59	III	1.77	0.95-3.32	0.069
		—	—	IV	—	—	—
S100Z	170591	29	26	I	0.45	0.17-1.21	.1
		114	60	II	1.24	0.72-2.12	.43
		84	34	III	0.54	0.25-1.17	.11
		—	—	IV	—	—	—

*P *< .05 was considered to be statistically significant.

HR, hazard ratio.

**Table 6.** Prognostic Value of mRNA Expression of S100 Family Members in Patients with Hepatocellular Carcinoma of Distinct Vascular Invasion

S100 Family	RNA-Seq ID	Number of Patients (n)		Vascular Invasion	HR	95% CI	*P*
		Low Expression	High Expression				
S100A1	6271	102	101	None	0.55	0.33-0.93	.023
		45	45	Micro	0.85	0.39-1.83	.67
		—	—	Macro	—	—	—
S100A2	6273	102	101	None	1.18	0.71-1.97	.52
		45	45	Micro	1.3	0.6-2.83	.50
		—	—	Macro	—	—	—
S100A3	6274	108	95	None	0.91	0.54-1.53	.72
		47	43	Micro	0.77	0.35-1.68	.51
		—	—	Macro	—	—	—
S100A4	6275	103	100	None	0.84	0.5-1.4	.5
		45	45	Micro	1.44	0.66-3.15	.36
		—	—	Macro	—	—	—
S100A5	6276	179	24	None	0.11	0.02-0.81	.0088
		77	13	Micro	0.18	0.02-1.32	.058
		—	—	Macro	—	—	—
S100A6	6277	102	101	None	0.92	0.55-1.54	.74
		45	45	Micro	1.11	0.51-2.41	.79
		—	—	Macro	—	—	—
S100A7	6278	170	33	None	1.22	0.61-2.41	.58
		70	20	Micro	3.13	1.42-6.87	.0029
		—	—	Macro	—	—	—
S100A7A	338824	195	8	None	1.57	0.57-4.35	.38
		85	5	Micro	0.52	0.07-3.87	.51
		—	—	Macro	—	—	—
S100A8	6279	103	100	None	0.63	0.37-1.06	.077
		45	45	Micro	1.37	0.63-2.98	.43
		—	—	Macro	—	—	—
S100A9	6280	102	101	None	1.12	0.67-1.86	.67
		45	45	Micro	2.23	1-4.97	.044
		—	—	Macro	—	—	—
S100A10	6281	102	101	None	1.16	0.69-1.94	.57
		45	45	Micro	1.99	0.89-4.43	.087
		—	—	Macro	—	—	—
S100A11	6282	102	101	None	1.13	0.68-1.88	.65
		45	45	Micro	1.38	0.63-3.02	.41
		—	—	Macro	—	—	—
S100A12	6283	107	96	None	0.72	0.43-1.2	.21
		52	38	Micro	0.55	0.24-1.27	.16
		—	—	Macro	—	—	—
S100A13	6284	102	101	None	1.14	0.69-1.91	0.61
		45	45	Micro	1.59	0.73-3.46	0.24
		—	—	Macro	—	—	—
S100A14	57402	102	101	None	1.22	0.73-2.05	.44
		45	45	Micro	0.85	0.4-1.81	.67
		—	—	Macro	—	—	—
S100A16	140576	102	101	None	1.09	0.65-1.82	.75
		45	45	Micro	1.14	0.53-2.46	.74
		—	—	Macro	—	—	—
S100B	6285	109	94	None	1.08	0.64-1.81	.77
		48	42	Micro	1.08	0.51-2.29	.85
		—	—	Macro	—	—	—
S100G	795	—	—	None	—	—	—
		—	—	Micro	—	—	—
		—	—	Macro	—	—	—
S100P	6286	102	101	None	1.27	0.76-2.13	.35
		45	45	Micro	2.11	0.95-4.71	.062
		—	—	Macro	—	—	—
S100PBP	64766	102	101	None	1.7	1-2.88	.047
		45	45	Micro	1.27	0.59-2.74	.55
		—	—	Macro	—	—	—
S100Z	170591	137	66	None	0.75	0.42-1.34	.34
		51	39	Micro	1.1	0.51-2.38	.81
		—	—	Macro	—	—	—

*P* < .05 was considered to be statistically significant.

HR, hazard ratio.
